# Expression of Neuropeptide FF Defines a Population of Excitatory Interneurons in the Superficial Dorsal Horn of the Mouse Spinal Cord that Respond to Noxious and Pruritic Stimuli

**DOI:** 10.1016/j.neuroscience.2019.08.013

**Published:** 2019-09-15

**Authors:** Maria Gutierrez-Mecinas, Andrew Bell, Erika Polgár, Masahiko Watanabe, Andrew J. Todd

**Affiliations:** a Institute of Neuroscience and Psychology, College of Medical, Veterinary and Life Sciences, University of Glasgow, Glasgow, G12 8QQ, UK; b Department of Anatomy, Hokkaido University School of Medicine, Sapporo 060-8638, Japan

**Keywords:** ALT, anterolateral tract, CCK, cholecystokinin, CTb, cholera toxin B subunit, DAPI, 4′,6-diamidino-2-phenylindole, eGFP, enhanced green fluorescent protein, ERK, extracellular signal-regulated kinases, GRP, gastrin releasing peptide, LPb, lateral parabrachial area, LSN, lateral spinal nucleus, NKB, neurokinin B, NPFF, neuropeptide FF, pERK, phospho-ERK, PKCγ, protein kinase Cγ isoform, NPFF, gastrin releasing peptide, neurokinin B, neurotensin, substance P, cholecystokinin

## Abstract

The great majority of neurons in the superficial dorsal horn of the spinal cord are excitatory interneurons, and these are required for the normal perception of pain and itch. We have previously identified 5 largely non-overlapping populations among these cells, based on the expression of four different neuropeptides (cholecystokinin, neurotensin, neurokinin B and substance P) and of green fluorescent protein driven by the promoter for gastrin-releasing peptide (GRP) in a transgenic mouse line. Another peptide (neuropeptide FF, NPFF) has been identified among the excitatory neurons, and here we have used an antibody against the NPFF precursor (pro-NPFF) and a probe that recognises Npff mRNA to identify and characterise these cells. We show that they are all excitatory interneurons, and are separate from the five populations listed above, accounting for ~ 6% of the excitatory neurons in laminae I-II. By examining phosphorylation of extracellular signal-regulated kinases, we show that the NPFF cells can respond to different types of noxious and pruritic stimulus. Ablation of somatostatin-expressing dorsal horn neurons has been shown to result in a dramatic reduction in mechanical pain sensitivity, while somatostatin released from these neurons is thought to contribute to itch. Since the great majority of the NPFF cells co-expressed somatostatin, these cells may play a role in the perception of pain and itch.

## Highlights

• NPFF is expressed by around 6% of the excitatory interneurons in the superficial dorsal horn of the mouse spinal cord.
• NPFF cells differ from those that express substance P, cholecystokinin, neurotensin or neurokinin B.
• Although some NPFF cells express gastrin-releasing peptide (GRP), they do not express GFP in a GRP-GFP mouse line.
• Some NPFF cells are activated by noxious or pruritic stimuli.

## **INTRODUCTION**

The superficial dorsal horn (laminae I-II) of the spinal cord receives excitatory synaptic input from primary sensory neurons that detect noxious, thermal and pruritic stimuli, and this information is conveyed to the brain *via* projection neurons belonging to the anterolateral tract (ALT) (Todd, 2010, Braz et al., 2014). Although the projection cells are concentrated in lamina I, they only account for ~ 1% of the neurons in the superficial dorsal horn (Abraira et al., 2017, Todd, 2017). The remaining nerve cells are defined as interneurons, and these have axons that remain within the spinal cord, where they contribute to local synaptic circuits (Peirs and Seal, 2016). Around 75% of the interneurons in laminae I-II are excitatory cells that use glutamate as their principal fast transmitter (Polgár et al., 2013). Behavioural assessment of mice in which excitatory interneurons in laminae I-II have been lost indicate that these cells are essential for the normal expression of pain and itch (Wang et al., 2013, Duan et al., 2014). However, the excitatory interneurons are heterogeneous in terms of their morphological, electrophysiological and neurochemical properties, and this has made it difficult to assign them to distinct functional populations (Todd, 2017).

We have identified 5 largely non-overlapping neurochemical populations among the excitatory interneurons in laminae I-II of the mouse spinal cord (Gutierrez-Mecinas et al., 2016, Gutierrez-Mecinas et al., 2017, Gutierrez-Mecinas et al., 2019). Cells belonging to 3 of these populations, which are defined by expression of neurotensin, neurokinin B (NKB, encoded by the Tac2 gene) and cholecystokinin (CCK), are concentrated in the inner part of lamina II, and extend into lamina III. These cells frequently co-express the γ isoform of protein kinase C (PKCγ). The other two populations consist of: (1) cells that express enhanced green fluorescent protein (eGFP) under control of the promoter for gastrin-releasing peptide (GRP) in a BAC transgenic mouse line (GRP-EGFP), and (2) cells that express the Tac1 gene, which codes for substance P (Dickie et al., 2019). The GRP-eGFP and substance P cells are located somewhat more dorsally than the other three populations, in the mid-part of lamina II. We have estimated that between them, these 5 populations account for around two-thirds of the excitatory interneurons in the superficial dorsal horn (Gutierrez-Mecinas et al., 2016, Gutierrez-Mecinas et al., 2017, Gutierrez-Mecinas et al., 2019). Our findings are generally consistent with the results of a recent transcriptomic study (Häring et al., 2018), which identified 15 clusters (named Glut1–15) among dorsal horn excitatory neurons. These included cells enriched with mRNAs for CCK (Glut1–3), neurotensin (Glut4), Tac2 (Glut5–7) and Tac1 (Glut10–11).

Another cluster identified by Häring et al. consisted of cells with mRNA for neuropeptide FF (NPFF; Glut9). Previous studies had identified NPFF-expressing cells in the superficial dorsal horn of rat spinal cord by using immunocytochemistry with anti-NPFF antibodies (Allard et al., 1991, Kivipelto and Panula, 1991). Both of these studies revealed a dense plexus of NPFF-immunoreactive axons in lamina I and the outer part of lamina II, which extended into the lateral spinal nucleus (LSN), together with scattered fibres in other regions including the intermediolateral cell column and the area around the central canal. Kivipelto and Panula (1991) also administered colchicine, which resulted in NPFF staining in cell bodies, and these were located throughout laminae I and II.

The aim of the present study was to identify and characterise NPFF-expressing cells in the mouse, by using a new antibody directed against the precursor protein pro-NPFF. In particular, our goal was to confirm that these were all excitatory interneurons and determine what proportion they accounted for, and to test the hypothesis that they formed a population that was distinct from those that we had previously identified. We also assessed their responses to different noxious and pruritic stimuli by testing for phosphorylation of extracellular signal-regulated kinases (ERK) (Ji et al., 1999).

## **Experimental Procedures**

### Animals

All experiments were approved by the Ethical Review Process Applications Panel of the University of Glasgow, and were performed in accordance with the European Community directive 86/609/EC and the UK Animals (Scientific Procedures) Act 1986.

We used three genetically modified mouse lines during the course of this study. One was the BAC transgenic Tg(GRP-EGFP) in which enhanced green fluorescent protein (eGFP) is expressed under control of the GRP promoter (Gong et al., 2003, Gutierrez-Mecinas et al., 2014, Solorzano et al., 2015). We have recently shown that virtually all eGFP-positive cells in this line possess GRP mRNA, although the mRNA is found in many cells that lack eGFP (Dickie et al., 2019). We also used a line in which Cre recombinase (fused to the ligand binding domain of the oestrogen receptor) is inserted into the *Grpr* locus (GRPR-iCreERT2) (Mu et al., 2017), and this was crossed with the Ai9 reporter line, in which Cre-mediated excision of a STOP cassette drives expression of tdTomato. Both GRP-EGFP and GRPR^CreERT2^; Ai9 mice were used for studies that assessed phosphorylated extracellular signal-regulated kinases (pERK) following noxious or pruritic stimuli. The use of GRPR^CreERT2^;Ai9 mice for some of these experiments allowed us also to assess responses of GRPR-expressing neurons, and this will be reported in a separate study.

Four adult wild-type C57BL/6 mice of either sex (18–25 g) and 3 adult GRP-EGFP mice of either sex (20–31 g) were deeply anaesthetised (pentobarbitone, 20 mg i.p.) and perfused through the left cardiac ventricle with fixative containing 4% freshly depolymerised formaldehyde in phosphate buffer. Lumbar spinal cord segments were removed and post-fixed for 2 h, before being cut into transverse sections 60 μm thick with a vibrating blade microtome (Leica VT1200, Leica Microsystems, Milton Keynes, UK). These sections were used for stereological analysis of the proportion of neurons that were pro-NPFF-immunoreactive, and also to look for the presence of pro-NPFF in GRP-eGFP, somatostatin-immunoreactive and Pax2-immunoreactive cells.

In order to determine whether any of the pro-NPFF-immunoreactive cells were projection neurons, we used tissue from 3 male wild-type C57BL/6 mice (32–35 g) that had received injections of cholera toxin B subunit (CTb) targeted on the left lateral parabrachial area (LPb) as part of previously published study (Cameron et al., 2015). In all cases, the CTb injection filled the LPb on the left side. Transverse sections from the L2 segments of these mice, which had been fixed as described above, were used for this part of the study.

To look for evidence that pro-NPFF cells responded to noxious or pruritic stimuli, we performed immunostaining for pERK (Ji et al., 1999) on tissue from GRPR^CreERT2^;Ai9 or GRP-EGFP mice. Twelve GRPR^CreERT2^;Ai9 female mice (17–23 g) were used to investigate responses to pinch or intradermally injected pruritogens, and in all cases, this was carried out under urethane anaesthesia (40–60 mg urethane i.p.). For 3 of the mice, five skin folds on the left calf were pinched for 5 s each (Dickie et al., 2019), and after 5 min, the mice were perfused with fixative as described above. The remaining mice received intradermal injections of histamine (100 μg/10 μl PBS; 3 mice), chloroquine (100 μg/10 μl PBS; 3 mice) or vehicle (10 μl PBS; 3 mice) into the left calf, which had been shaved the day before. The success of the intradermal injections was verified by the presence of a small bleb in the skin (Roberson et al., 2013). We have previously shown that intradermal injections of vehicle result in pERK labelling if mice are allowed to survive for 5 mins after the stimulus, probably due to the noxious mechanical stimulus resulting from i.d. injection (Bell et al., 2016). However, if the mice survive 30 mins, pERK is seen in pruritogen-injected, but not vehicle-injected animals, and this presumably reflects prolonged activation by the pruritogens. We therefore waited until 30 mins after the injections before intracardiac perfusion with fixative, which was carried out as described above. Tissue from 6 urethane-anaesthetised GRP-EGFP mice that had had the left hindlimb immersed in water at 52 °C for 15 s or had received a subcutaneous injection of capsaicin (10 μl, 0.25%) was also used. In these cases, the tissue was obtained from experiments that had formed part of a previously published study (Dickie et al., 2019), and injection of the vehicle for capsaicin had been shown to result in little or no pERK labelling. Capsaicin had initially been prepared at 1% by dissolving it in a mixture of 7% Tween 80 and 20% ethanol in saline. It was then diluted to 0.25% before injection.

Fluorescent *in situ* hybridisation was performed on lumbar spinal cord sections from 3 C57BL/6 mice (either sex, 18-20 g), tissue from which had been used in a previous study (Gutierrez-Mecinas et al., 2019).

### Immunocytochemistry, confocal scanning and analysis

Multiple-labelling immunofluorescence reactions were performed as described previously (Gutierrez-Mecinas et al., 2017) on 60 μm thick transverse sections of spinal cord. The sources and concentrations of antibodies used are listed in Table 1. Sections were incubated for 3 days at 4 °C in primary antibodies diluted in PBS that contained 0.3 M NaCl, 0.3% Triton X-100 and 5% normal donkey serum, and then overnight in appropriate species-specific secondary antibodies (Jackson Immunoresearch, West Grove, PA) that were raised in donkey and conjugated to Alexa 488, Alexa 647, Rhodamine Red or biotin. All secondary antibodies were used at 1:500 (in the same diluent), apart from those conjugated to Rhodamine Red, which were diluted to 1:100. Biotinylated secondary antibodies were detected with Pacific Blue conjugated to avidin (1:1000; Life Technologies, Paisley, UK). Following the immunocytochemical reaction, sections were mounted in anti-fade medium and stored at − 20 °C.

**Table 1:** Antibodies used in this study.

Antibody	Species	Catalogue no	Dilution	Source
pro-NPFF	Guinea pig		0.83 μg/ml	M Watanabe
NeuN	Mouse	MAB377	1:500	Merck
eGFP	Chicken	ab13970	1:1000	Abcam
Pax2	Rabbit	HPA047704	1:200	Sigma
Somatostatin	Rabbit	T-4103	1:1000	Peninsula
CTb	Goat	703	1:1000	List Biological
pERK	Rabbit	9101	1:500	Cell Signalling Technology

Sections from 3 wild-type mice were reacted with the following combinations of primary antibodies: (1) pro-NPFF and NeuN; (2) pro-NPFF, somatostatin and NeuN. Those reacted with the first combination were subsequently stained with the nuclear stain 4′6-diamidino-2-phenylindole (DAPI). Sections from 3 GRP-EGFP mice were reacted with the following combination: pro-NPFF, eGFP, Pax2 and NeuN. Sections from mice that had received injection of CTb into the LPb were reacted with antibodies against pro-NPFF, CTb and NeuN. Sections from mice that had undergone the various types of noxious or pruritic stimulation were reacted with antibodies against pro-NPFF, pERK and NeuN.

Sections were scanned with a Zeiss LSM 710 confocal microscope with Argon multi-line, 405 nm diode, 561 nm solid state and 633 nm HeNe lasers. Confocal image stacks were obtained through a 40 × oil immersion lens (numerical aperture 1.3) with the confocal aperture set to 1 Airy unit, and unless otherwise stated, the entire mediolateral width of laminae I-II was scanned to generate z-series of at least 20 μm (and in many cases the full thickness of the section), with a z-separation of 1 μm. Confocal scans were analysed with Neurolucida for Confocal software (MBF Bioscience, Williston, VT). The lamina II-III border was identified from the distribution of NeuN immunoreactivity, based on the relatively low neuronal packing density in lamina IIi. The lamina I-II border was assumed to be 20 μm from the dorsal edge of the dorsal horn (Ganley et al., 2015).

To determine the proportion of neurons in laminae I-II that are pro-NPFF-immunoreactive, we used a modification of the optical disector method (Polgár et al., 2004) on 2 sections each from 3 wild-type mice reacted with the first combination of antibodies. The reference and look-up sections were set 10 μm apart, and initially only the NeuN and DAPI channels were viewed. All intervening optical sections were examined, and neuronal nuclei (NeuN +/DAPI + structures) were selected if their bottom surface lay between the reference and look-up sections. These were plotted onto an outline drawing of the dorsal horn. The pro-NPFF channel was then switched on and the presence or absence of staining was determined for each of the selected neurons. To estimate the extent of co-localisation of NPFF and somatostatin, we scanned 2 sections that had been reacted with the 2nd antibody combination from each of 3 wild-type mice. The pro-NPFF and NeuN channels were viewed, and all pro-NPFF cells throughout the full thickness of the section were identified. The somatostatin channel was then switched on, and the presence or absence of somatostatin in each selected cell was noted. We searched for overlap between pro-NPFF and eGFP or Pax2 in two sections each from three GRP-EGFP mice. Again, all pro-NPFF-immunoreactive cells throughout the depth of the section were initially identified, and the presence or absence of eGFP and Pax2 was then determined. To test whether any of the pro-NPFF cells were projection neurons, we scanned and analysed between 4 and 7 sections from each of 3 mice that had received CTb injections into the LPb (Cameron et al., 2015). We identified all lamina I CTb + cells that were visible within each section and checked for the presence of pro-NPFF-immunoreactivity.

Analysis of ERK phosphorylation in pro-NPFF cells was performed as described previously (Bell et al., 2016, Gutierrez-Mecinas et al., 2017, Dickie et al., 2019). Sections that contained numerous pERK + cells were initially selected and scanned with the confocal microscope through the 40 × oil-immersion lens to generate z-stacks (1 μm z-separation) through the full thickness of the section so as to include the region of dorsal horn that contained pERK cells. The outline of the dorsal horn, together with the lamina II/III border, was plotted with Neurolucida, and the mediolateral extent of the region that contained a high density of pERK cells was delineated by drawing two parallel lines that were orthogonal to the laminar boundaries. The channels corresponding to NeuN and pro-NPFF were initially viewed, and all pro-NPFF + cells within this region were plotted onto the drawing. The pERK channel was then viewed, and the presence or absence of staining in each of the selected pro-NPFF cells was noted.

### Fluorescent in situ hybridization histochemistry

Multiple-labelling fluorescent *in situ* hybridisation was performed with RNAscope probes and RNAscope fluorescent multiplex reagent kit 320,850 (ACD BioTechne; Newark, CA 94560). Fresh frozen lumbar spinal cord segments from 3 wild-type mice were embedded in OCT mounting medium and cut into 12 μm thick transverse sections with a cryostat (Leica CM1950; Leica; Milton Keynes, UK). These were mounted non-sequentially (such that sections on the same slide were at least 4 apart) onto SuperFrost Plus slides (48311–703; VWR; Lutterworth, UK) and air dried. Reactions were carried out according to the manufacturer's recommended protocol. The probes used in this study, and the proteins/peptides that they correspond to, are listed in Table 2. Sections from 3 mice were incubated in the following probe combinations: (1) Npff, Grp, Tac1; (2) Npff, Cck, Tac2; (3) Npff, Nts. Probes were revealed with Alexa 488 (Npff), Atto 550 (Grp, Cck) and Alexa 647 (Tac1, Tac2 and Nts). Sections were mounted with Prolong-Glass anti-fade medium with NucBlue (Hoechst 33342) (ThermoFisher Scientific, Paisley, UK). Positive and negative control probes were also tested on other sections (Table 2).

**Table 2:** RNAscope probes.

Probe	Protein/peptide	Channel numbers	Catalogue numbers	Z-pair number	Target region
Npff	Neuropeptide FF	2	479,901	9	47–433
Cck	CCK	1	402,271	12	23–679
Grp	Gastrin-releasing peptide	1	317,861	15	22–825
Nts	Neurotensin	3	420,441	20	2–1188
Tac1	Substance P	3	410,351	15	20–1034
Tac2	NKB	3	446,391	15	15–684
RNAscope multiplex positive control (Polr2a, Ppib, Ubc)	Polr2a: DNA-directed RNA polymerase II subunit RPB1; Ppib: Peptidyl-prolyl cis-trans isomerase B; Ubc: Polyubiquitin-C	1,2,3	320,881	20 15 n/a	2802–3678 98–856 34–860
RNAscope multiplex negative control (dapB)	dapB: 4-hydroxy-tetrahydrodipicolinate reductase (derived from B Subtilis)	1,2,3	320,871	10	414–862

Sections were scanned with a Zeiss LSM 710 confocal microscope as above. Since the sections reacted with each probe combination were obtained from a 1 in 4 series, there was at least 36 μm separation between the scanned sections. Confocal image stacks were obtained through the 40 × oil immersion lens with the confocal aperture set to 1 Airy unit, and the entire mediolateral width of laminae I-II was scanned to generate a z-series of the full thickness of the section, with a z-separation of 2 μm. Confocal scans of 5 sections per animal were analysed with Neurolucida for Confocal software. Initially, only the channel corresponding to Npff mRNA was examined and all Npff positive NucBlue nuclei were identified. Then channels corresponding to other probes were viewed and any co-localisation noted. Cells were defined as positive for a particular mRNA if greater than 4 transcripts were present in the nucleus or immediate perinuclear area.

### Characterization of antibodies

**Fig. 1:**
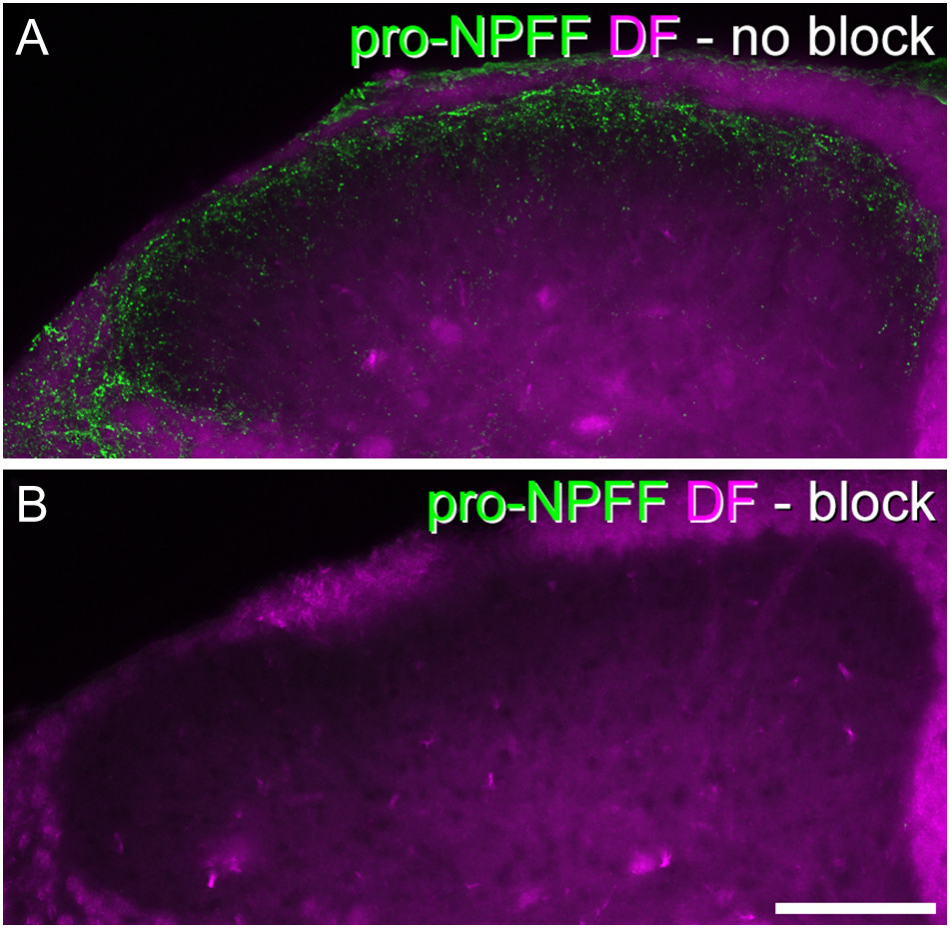
Blocking of pro-NPFF immunostaining by pre-absorption with the antigen. A: transverse section from the L3 segment of a mouse, reacted with pro-NPFF antibody. Pro-NPFF-immunoreactivity (green) is superimposed on a dark-field image (magenta). B: A section reacted with pro-NPFF antibody that had been pre-absorbed overnight with 4.4 μg/ml of antigen, and scanned under identical conditions. The immunoreactivity has been completely abolished. Both images are maximum intensity projections of 11 optical sections at 2 μm z-spacing. Scale bar = 100 μm. (For interpretation of the references to colour in this figure legend, the reader is referred to the web version of this article.)

The sources and dilutions of primary antibodies used in the study are listed in Table 1. The pro-NPFF antibody was raised against a fusion protein consisting of glutathione S-transferase and amino acids 22–114 of the mouse pro-NPFF protein (NCBI, NM_018787). Staining was completely abolished by pre-incubating the antibody at its normal working concentration (0.83 μg/ml) with the antigen at 4.4 μg/ml (Fig. 1). The mouse monoclonal antibody NeuN reacts with a protein in cell nuclei extracted from mouse brain (Mullen et al., 1992), which has subsequently been identified as the splicing factor Fox-3 (Kim et al., 2009). This antibody apparently labels all neurons but no glial cells in the rat spinal dorsal horn (Todd et al., 1998). The eGFP antibody was raised against recombinant full-length eGFP, and its distribution matched that of native eGFP fluorescence. The Pax2 antibody was raised against amino acids 268–332 of the human protein, and it has been shown that this labels essentially all GABAergic neurons in adult rat dorsal horn (Larsson, 2017). The somatostatin antiserum is reported to show 100% cross-reactivity with somatostatin-28 and somatostatin-25, but none with substance P, neuropeptide Y, or vasoactive intestinal peptide (manufacturer's specification), and we have shown that staining with this antibody is abolished by pre-incubation with 10 μg/ml somatostatin (Proudlock et al., 1993). The CTb antibody was raised against the purified protein, and specificity is demonstrated by the lack of staining in regions that did not contain injected or transported tracer. The pERK antibody detects p44 and p42 MAP kinase (Erk1 and Erk2) when these are phosphorylated either individually or dually at Thr202 and Tyr204 of Erk1 or Thr185 and Tyr187 of Erk2. This antibody does not cross-react with the corresponding phosphorylated residues of JNK/SAPK or of p38 MAP kinase, or with non-phosphorylated Erk1/2 (manufacturer's specification). Specificity is demonstrated by the lack of staining in non-stimulated areas (*e.g.* in the contralateral dorsal horn).

## **RESULTS**

### Pro-NPFF-immunoreactivity in the dorsal horn

**Fig. 2:**
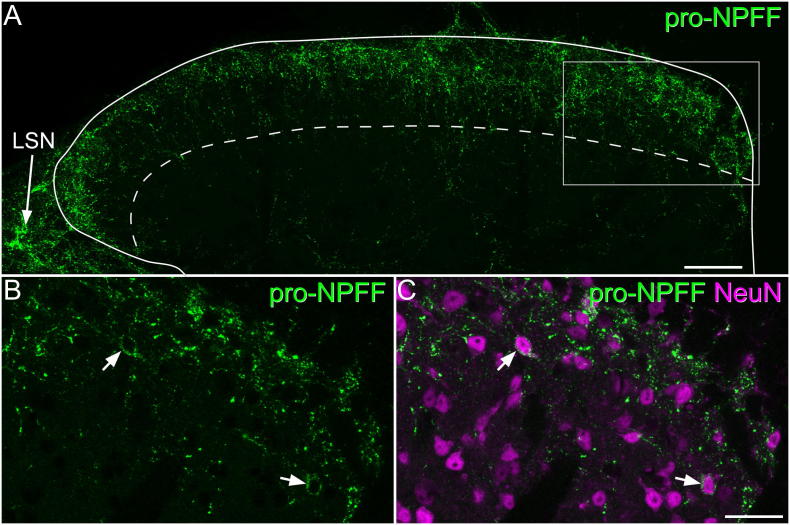
Pro-NPFF-immunoreactivity in the dorsal horn and lateral spinal nucleus. A: transverse section through the L4 spinal segment of a mouse reacted with antibody against pro-NPFF (green). The dorsal horn outline is shown with a solid line and the lamina II-III border with a dashed line. There is a high density of immunoreactive axons in lamina I and the outer part of lamina II, with scattered profiles in deeper laminae. There is also a dense plexus in the lateral spinal nucleus (LSN) (arrow). The box indicates the area shown in B and C. B, C: a higher magnification view showing the relation of pro-NPFF (green) to NeuN (magenta). Two immunoreactive cell bodies are indicated with arrows. Note that the pro-NPFF immunostaining occupies the perikaryal cytoplasm, surrounding the nucleus (which is labelled strongly with the NeuN antibody). The images are maximum intensity projection of 23 optical sections (A) or 3 optical sections (B,C) at 1 μm z-separation. Scale bars: A = 50 μm; B,C = 25 μm. (For interpretation of the references to colour in this figure legend, the reader is referred to the web version of this article.)

Immunoreactivity for pro-NPFF was highly concentrated in the superficial dorsal horn (laminae I-II) and the LSN, with a distribution very similar to that reported previously in the rat with antibodies against NPFF (Allard et al., 1991, Kivipelto and Panula, 1991) (Fig. 2). At high magnification, most immunoreactive profiles resembled axon terminals, but there were also labelled cell bodies, in which the immunoreactivity was present in the perikaryal cytoplasm (Fig. 2B and C). These pro-NPFF-immunoreactive cell bodies were present throughout laminae I-II, but were most numerous in the dorsal half of this region. They were not seen in the LSN.

**Fig. 3:**
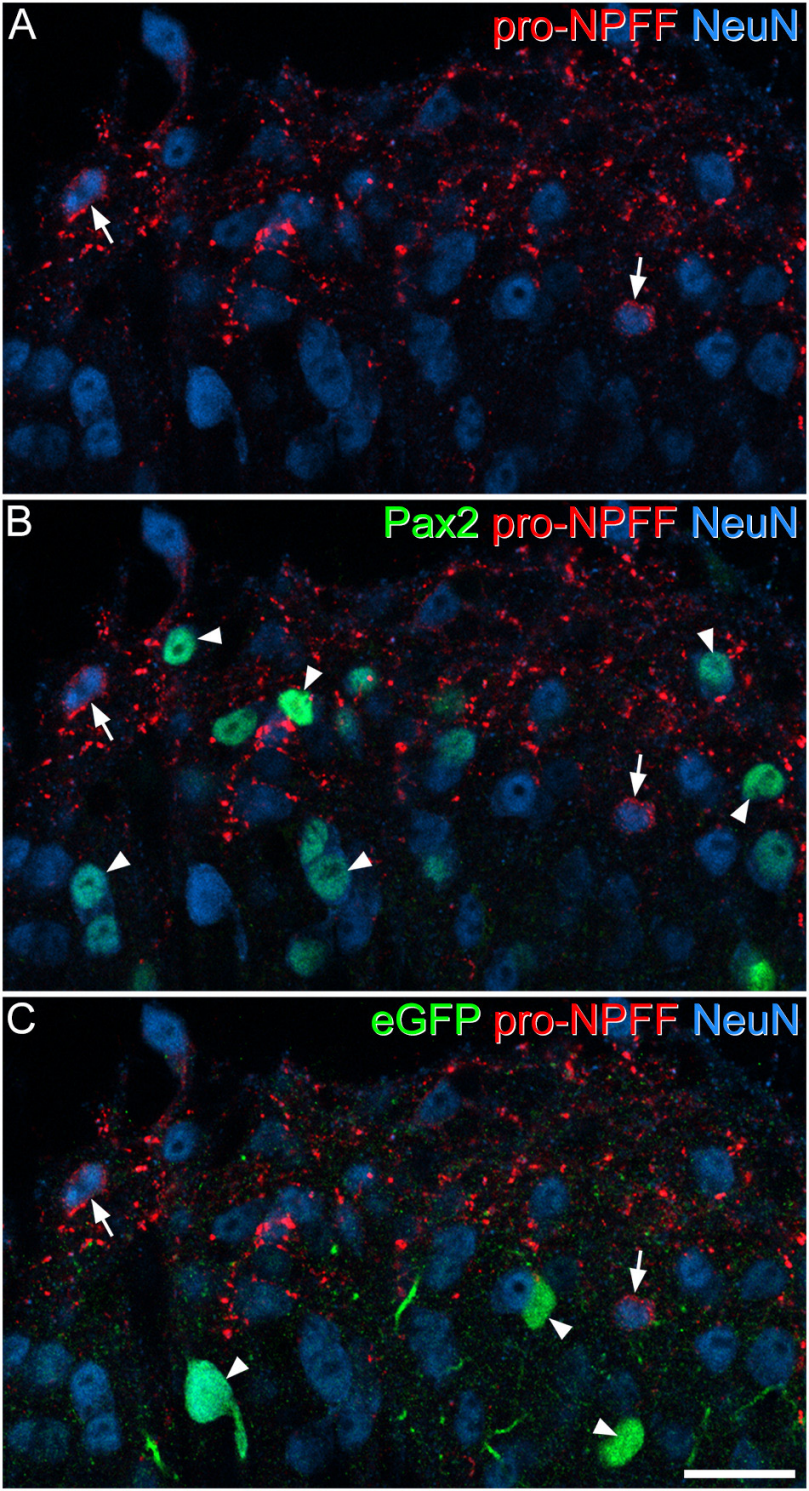
Lack of co-localisation of pro-NPFF with either Pax2 or eGFP in the GRP-EGFP mouse. Part of the superficial dorsal horn of a GRP-EGFP mouse immunostained for pro-NPFF (red) and NeuN (blue), together with Pax2 and eGFP (both of which are shown in green, in B and C, respectively). A: Two pro-NPFF-immunoreactive cells are shown with arrows. These cells are negative for both Pax2 (B) and eGFP (C). Arrowheads in B and C show cells that are Pax2-immunoreactive (B) or eGFP-immunoreactive (C). Images are projections of 2 optical sections at 1 μm z-spacing. Scale bar = 20 μm. (For interpretation of the references to colour in this figure legend, the reader is referred to the web version of this article.)

**Fig. 4:**
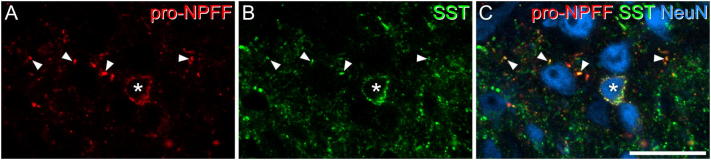
Co-localisation of pro-NPFF and somatostatin in the superficial dorsal horn. A,B: Part of lamina II scanned to reveal pro-NPFF (red) and somatostatin (SST, green). C: the same field scanned to reveal both peptides, together with NeuN (blue). The asterisk shows a cell that contains both pro-NPFF and somatostatin in its perikaryal cytoplasm, and therefore expresses both peptides. Arrowheads point to varicosities that contain both types of peptide immunoreactivity, and presumably correspond to axon terminals. The image is from a single optical section. Scale bar = 20 μm. (For interpretation of the references to colour in this figure legend, the reader is referred to the web version of this article.)

In the quantitative analysis with the disector technique, we identified a mean of 392 (range 323–444) NeuN-positive cells in laminae I-II per mouse (n = 3 mice), and found that 4.74% (3.83–6.36%) of these were pro-NPFF-immunoreactive. To test the prediction that pro-NPFF cells were excitatory (Häring et al., 2018), we looked for the presence of Pax2. This was carried out on tissue from the GRP-EGFP mouse, which also allowed us to determine whether there was any co-expression of pro-NPFF and eGFP. We identified a mean of 55.6 pro-NPFF cells in this tissue (50–66, n = 3 mice), and found that none of these were either Pax2- or eGFP-immunoreactive (Fig. 3). Somatostatin is expressed by many excitatory interneurons in the superficial dorsal horn (Gutierrez-Mecinas et al., 2016), and we therefore looked for co-localisation of pro-NPFF- and somatostatin-immunoreactivity. We identified 62.7 (56–67) pro-NPFF-immunoreactive cells in sections from 3 wild-type mice, and found that 85.3% (81.5–89.3%) of these were also immunoreactive for somatostatin (Fig. 4). There was also extensive co-localisation of pro-NPFF and somatostatin in axonal boutons.

### Fluorescence in situ hybridization

**Fig. 5:**
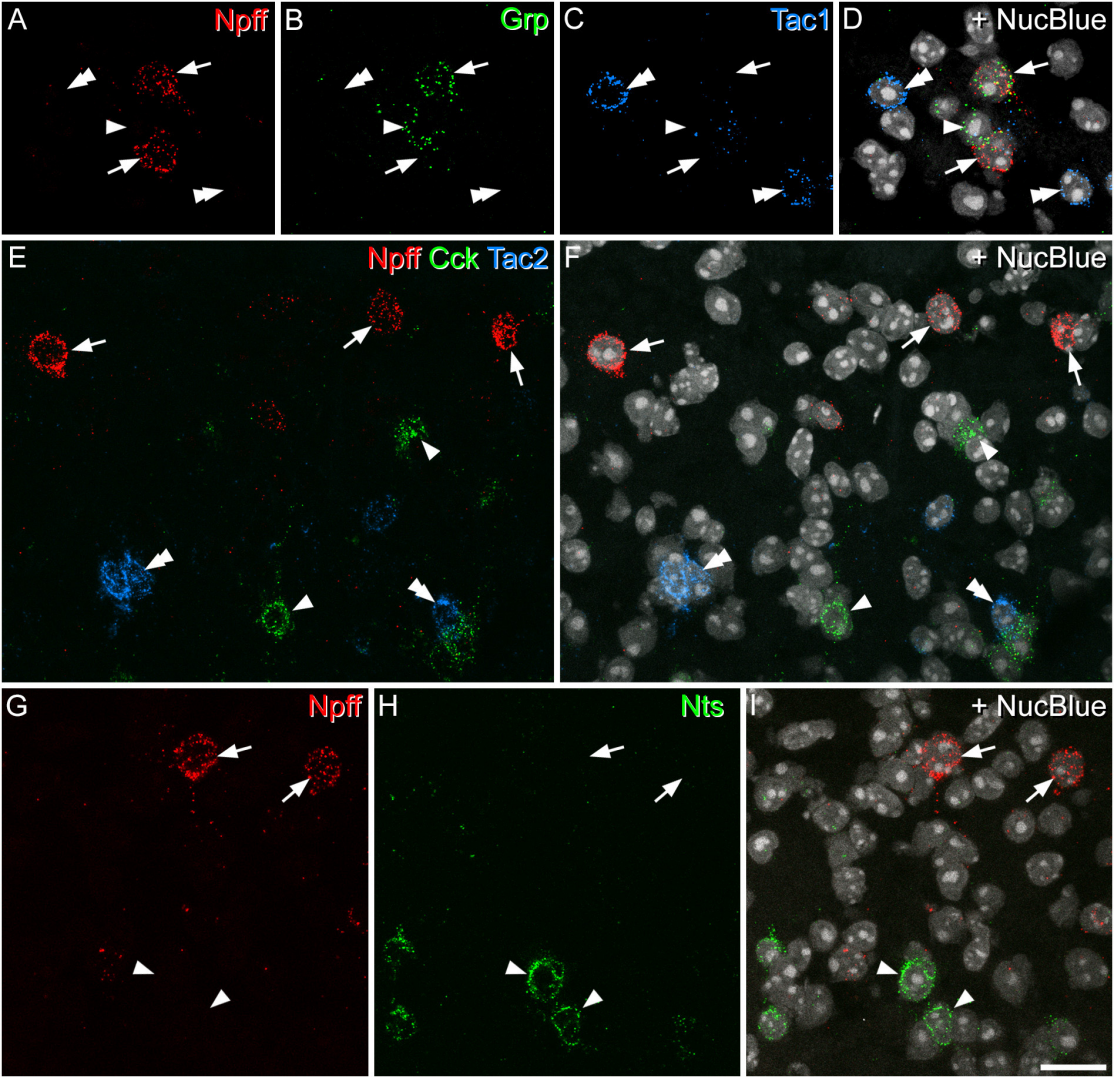
Fluorescence *in situ* hybridisation for Npff and other neuropeptides. A-C show part of lamina II in a section reacted with probes for Npff (red), Grp (green) and Tac1 (blue) mRNAs. D is the same field, which has been scanned to reveal the nuclear counterstain NucBlue (grey). Two Npff mRNA + cells are indicated with arrows. These are both positive for Grp mRNA, but negative for Tac1 mRNA. Another cell with Grp mRNA only is shown with a single arrowhead, and two Tac1 mRNA + cells (which lack Npff and Grp mRNAs) are indicated with double arrowheads. E: part of laminae I and II scanned to reveal mRNAs for Npff (red), Cck (green) and Tac2 (blue). F: the same section also showing NucBlue (grey). Three Npff mRNA + cells are indicated with arrows, and these lack mRNAs for Cck or Tac2. Examples of cells with mRNA for Cck and Tac2 are shown with single and double arrowheads, respectively. G,H show part of laminae I and II scanned to reveal mRNAs for Npff (red) and Nts (green). I: The same field also showing NucBlue (grey). Two Npff mRNA + cells are indicated with arrows, and two Nts mRNA + cells with arrowheads. All images were projected from 2 optical sections at 2 μm z-separation. Scale bar = 20 μm. (For interpretation of the references to colour in this figure legend, the reader is referred to the web version of this article.)

The distribution of cells that contained Npff mRNA was the same as that of cells with pro-NPFF immunoreactivity. They were largely restricted to the superficial dorsal horn, and were most numerous in lamina I and the outer part of lamina II. In sections reacted with probes against Npff, Tac1 and Grp mRNAs, we identified 58.7 (51–65) Npff mRNA + cells in tissue from each of 3 mice. We found very limited overlap with Tac1, since only 4.6% (3.3–5.9%) of these cells were also Tac1 mRNA + (Fig. 5A, C, D). However, there was extensive overlap with the mRNA for Grp (Fig. 5A, B, D). Grp mRNA was found in 38% (28–47%) of Npff mRNA + cells, and this represented 6.3% (5.3–7.3%) of the Grp mRNA + cells in laminae I-II. In the sections reacted for Npff, Cck and Tac2 mRNAs, we identified 57 (53–62) Npff mRNA + cells from the 3 mice. None of these were positive for Tac2 mRNA, and only one was positive for Cck mRNA (corresponding to 0.6% of the Npff population) (Fig. 5E and F). In sections reacted for Npff and Nts mRNAs, we found 60.7 (56–68) Npff mRNA + cells in the 3 mice (Fig. 5G-I). Only two of these cells were Nts mRNA + (corresponding to 1% of the Npff population).

### Lack of labelling of PNs

**Fig. 6:**
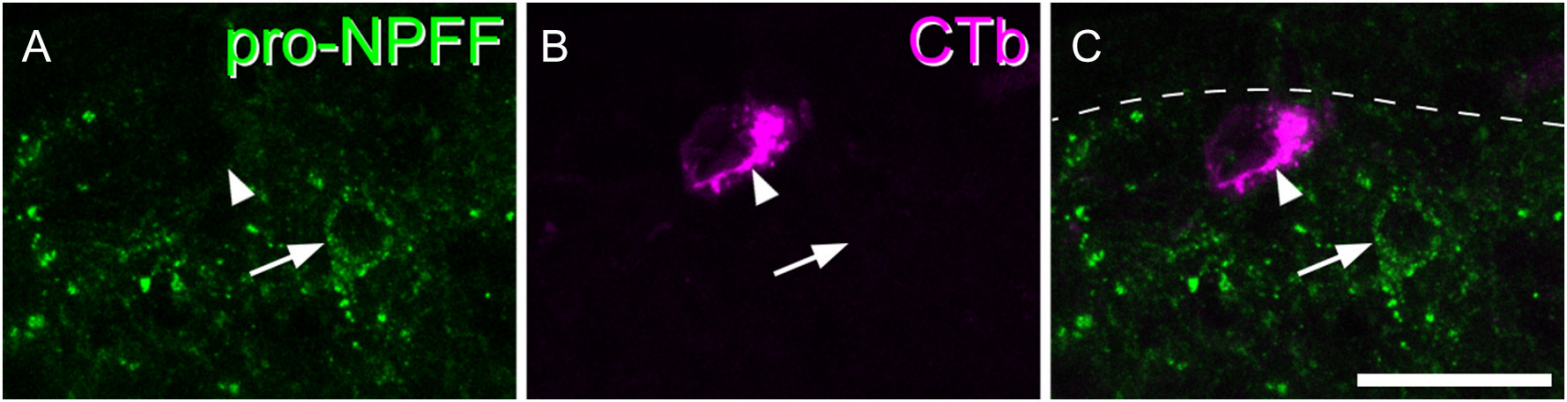
Lack of pro-NPFF in lamina I projection neurons. A,B: part of lamina I from a mouse that had received an injection of cholera toxin B subunit (CTb) into the lateral parabrachial area on the contralateral side. A cell with pro-NPFF-immunoreactivity is indicated with an arrow, and a nearby CTb-labelled lamina I projection neuron with an arrowhead. C: merged image showing the two cells. The dashed line represents the dorsal border of lamina I. The image is a projection of 3 optical sections at 1 μm z-separation. Scale bar = 20 μm.

We identified a total of 111 CTb-labelled neurons in lamina I in the 3 animals that had received injections into the LPb (29–52 per mouse). None of the CTb-labelled cells were pro-NPFF-immunoreactive (Fig. 6).

### pERK

**Fig. 7:**
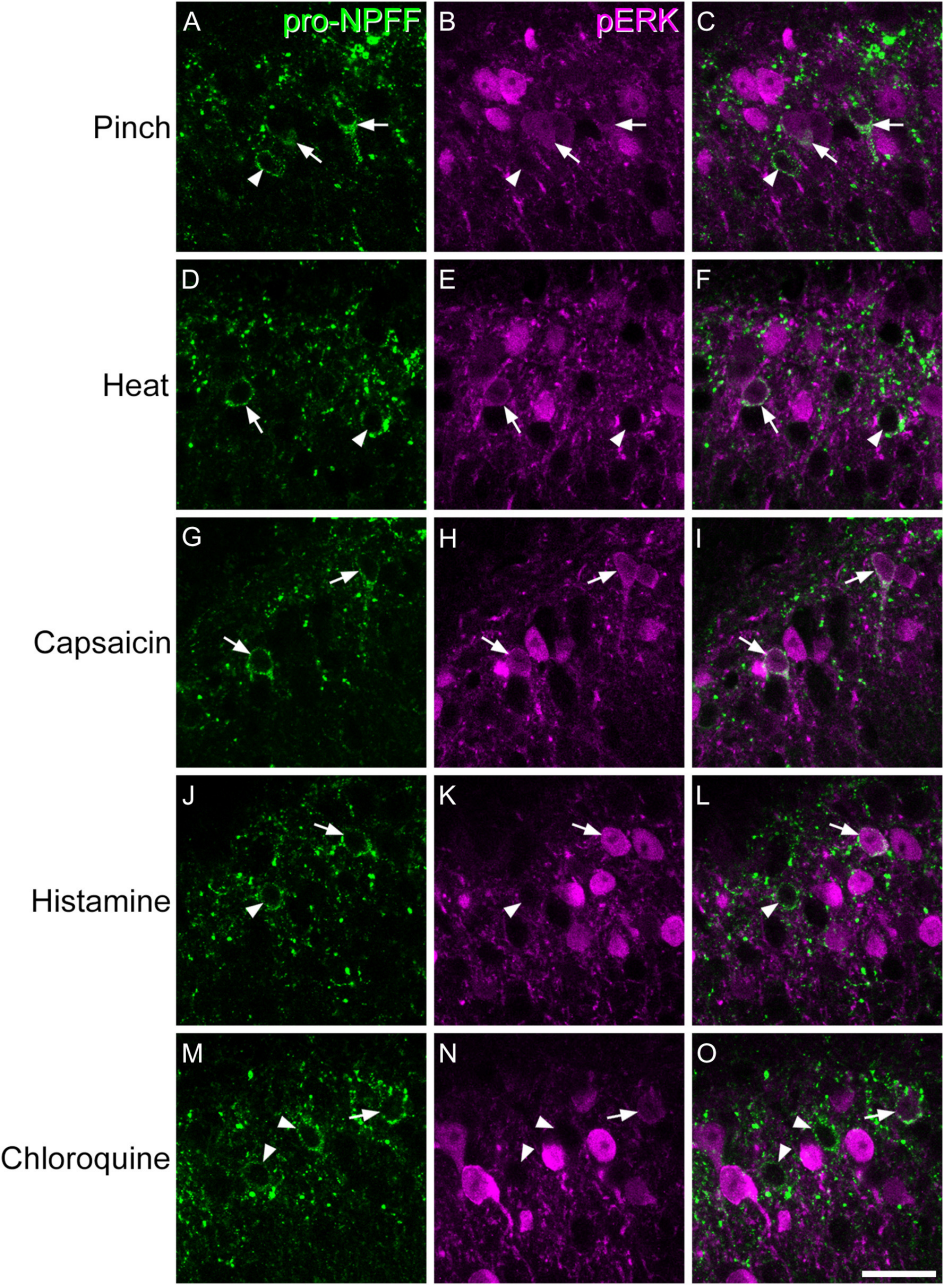
Phosphorylation of extracellular signal-regulated kinases in NPFF-expressing neurons. Each row shows part of the superficial dorsal horn immunostained for pro-NPFF (green; A,D,G,J,M), pERK (magenta; B,E,H,K,N) together with a merged image (C,F,I,L,O), following pinch (A-C), noxious heat (D-F), intraplantar capsaicin (G-I), intradermal histamine (J-L) or intradermal chloroquine (M-O). Pro-NPFF cells that are pERK + are indicated with arrows, and those that lack pERK with arrowheads. Note that some of the pro-NPFF cells show relatively weak pERK immunoreactivity. Images are from single optical sections (G-I) or from maximum projections of 2 (A-C, J-L, M-O) or 3 (D-F) optical sections at 1 μm z-separation. Scale bar = 20 μm. (For interpretation of the references to colour in this figure legend, the reader is referred to the web version of this article.)

The distribution of pERK-immunoreactivity in mice that had received noxious or pruritic stimuli was very similar to that described previously (Ji et al., 1999, Bell et al., 2016, Dickie et al., 2019). In each case, pERK-positive cells were only seen on the side ipsilateral to the stimulus, in the somatotopically appropriate region of the dorsal horn, and they were most numerous in the superficial laminae (I-II) (Fig. 7). Few, if any, pERK + cells were seen in mice that had received intradermal injection of vehicle. For each of the stimuli examined, we found that some pro-NPFF-immunoreactive cells were pERK-positive (Fig. 7, Table 3), although in many cases these cells showed relatively weak pERK immunoreactivity (Fig. 7C, F, O). For the noxious heat stimulus, and for both pruritogens (chloroquine, histamine), the proportion of pro-NPFF cells with pERK was around 30%, while for pinch and capsaicin injection the proportions were higher (around 50% and 60%, respectively; Table 3).

**Table 3:** NPFF cells that were pERK positive following noxious or pruritic stimuli.

Stimulus	NPFF^+^ neurons	NPFF^+^ and pERK^+^	% NPFF neurons pERK^+^	% of all neurons in laminae I-II with pERK
Pinch	82.3 (71–92)	41.6 (26–50)	49.8% (36.6–58.3%)	37%
Noxious heat	104.7 (102–110)	31.7 (27–39)	30.1% (26.5–35.5%)	23%
Capsaicin	90 (80–102)	55.7 (50–63)	62% (56.8–67.5%)	27%
Histamine	91 (76–109)	30 (28–34)	33.3% (31.5–36.8%)	33%
Chloroquine	114.3 (95–132)	37.7 (27–56)	32.2% (25.9–42.4%)	21%*

Column 2–4 show the number of NPFF cells identified within the zone that showed pERK, the number of NPFF/pERK double-labelled cells, and the proportion of NPFF cells with pERK, respectively. The 5th column shows the proportion of all neurons in laminae I-II that were found to be pERK-immunoreactive with equivalent stimuli in previous studies (Bell et al., 2016, Dickie et al., 2019). *Note that the proportion of laminae I-II neurons that were pERK + after chloroquine (21%) was estimated in experiments involving a lower dose of chloroquine (40 μg) than that used in the present study (100 μg) (Bell et al., 2016).

## **DISCUSSION**

Our main findings are that the pro-NPFF antibody labels a population of excitatory interneurons in laminae I-II, that these cells account for nearly 5% of all neurons in this region, that they are distinct from cells belonging to other neurochemical populations that have recently been defined (Gutierrez-Mecinas et al., 2016, Gutierrez-Mecinas et al., 2017, Gutierrez-Mecinas et al., 2019), and that many of them respond to noxious and/or pruritic stimuli.

### Technical considerations

The laminar pattern of staining with the pro-NPFF antibody closely matched that described previously for NPFF antibodies in rat spinal cord (Allard et al., 1991, Kivipelto and Panula, 1991), while the immunoreactive cell bodies showed a similar distribution to that seen with *in situ* hybridisation using NPFF probes. In addition, the lack of expression in Pax2-positive cells is consistent with the restriction of NPFF to excitatory neurons (Häring et al., 2018). Taken together with the finding that pre-absorption with the antigen blocked immunostaining, these observations suggest that this new antibody was indeed detecting NPFF-expressing neurons. Further confirmation of its specificity could be obtained in the future by testing the antibody on tissue from NPFF knock-out mice.

### NPFF cells as a distinct population of excitatory interneurons

Earlier immunocytochemical and *in situ* hybridisation studies have shown a relatively high density of NPFF-expressing cells and processes in the superficial dorsal horn of the rodent spinal cord (Allard et al., 1991, Kivipelto and Panula, 1991, Lee et al., 1993, Panula et al., 1999, Vilim et al., 1999, Häring et al., 2018), with a distribution that closely matched that seen with the pro-NPFF antibody and the Npff mRNA probe in the present study. Häring et al. (2018) showed that these cells, which mainly represented their Glut9 cluster, co-expressed the mRNA for Slc17a6 (which codes for VGLUT2), indicating that these were excitatory neurons. This accords with our finding that pro-NPFF-expressing cells were invariably negative for Pax2-immunoreactivity, which is present in all inhibitory neurons in the dorsal horn (Cheng et al., 2004, Larsson, 2017).

None of the pro-NPFF-immunoreactive cells were retrogradely labelled with CTb that had been injected into the lateral parabrachial area, and since tracer injections into this region are thought to label virtually all projection neurons in lamina I (Todd, 2010, Cameron et al., 2015), it is likely that all of the NPFF cells are excitatory interneurons. We have previously reported that 76% of neurons in the mouse superficial dorsal horn are excitatory (Pax2-negative) (Polgár et al., 2013), and since we found pro-NPFF-immunoreactivity in 4.7% of lamina I-II neurons, we estimate that the NPFF-expressing cells account for around 6% of all excitatory neurons in this region. Our finding that the NPFF population showed virtually no overlap with those defined by expression of Cck, Nts or Tac2, and only minimal overlap with the Tac1 population is consistent with the findings of Haring et al., since these cells would correspond to those belonging to Glut1–3 (Cck), Glut4 (Nts), Glut5–7 (Tac2) and Glut10–11 (Tac1). We have also examined sections that had been immunostained for pro-NPFF together with various combinations of antibodies against neurotensin, preprotachykinin B (the precursor for NKB) and pro-cholecystokinin (the precursor for CCK), and found no overlap of any of these with pro-NPFF (M Gutierrez-Mecinas and AJ Todd, unpublished data). With regard to GRP, there was an apparent discrepancy, since we found no overlap between pro-NPFF-immunoreactivity and eGFP expression in the GRP-EGFP mouse, but nearly 40% of the Npff mRNA + cells also contained Grp mRNA. By using multiple-label fluorescent *in situ* hybridisation we have found that although all Gfp mRNA + cells in this mouse line contain Grp mRNA, these only account for around 25% of the Grp mRNA + cells (AM Bell, unpublished observations). Interestingly, Häring et al. reported that Grp mRNA was widely distributed among several excitatory interneuron clusters (Glut5–12), whereas we have found that the GRP-eGFP-positive cells in lamina II form a relatively homogeneous population that shows very little overlap with neurons that express CCK, neurotensin, substance P or NKB (Gutierrez-Mecinas et al., 2016, Gutierrez-Mecinas et al., 2019. Dickie et al., 2019). In addition, these cells show a unique somatotopic distribution, as they are far less frequent in regions that are innervated from glabrous skin (Dickie et al., 2019). This suggests that the GRP-eGFP cells represent a discrete functional population, even though Grp message is far more widely expressed.

**Fig. 8:**
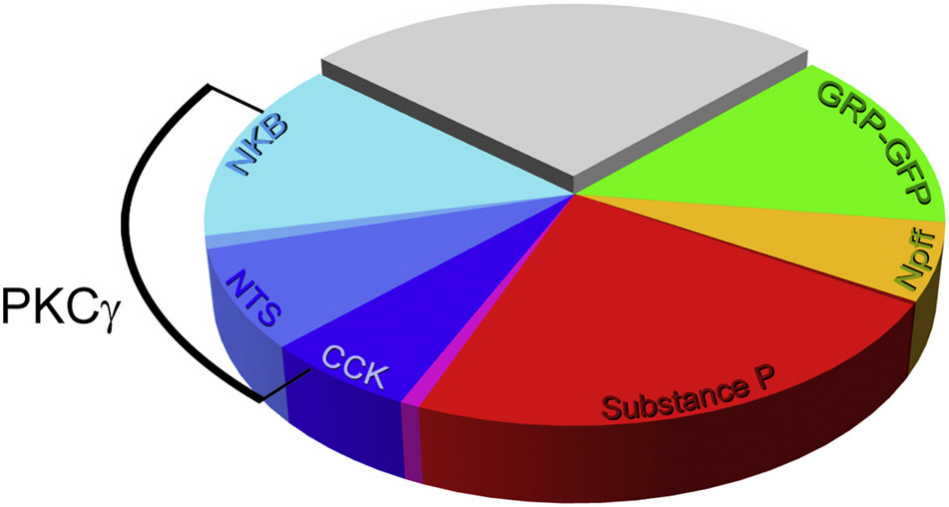
Pie chart showing the relative sizes of different neurochemical populations among the excitatory interneurons in laminae I-II of the mouse dorsal horn. Note that the neurokinin B (NKB), neurotensin (NTS), cholecystokinin (CCK), substance P, NPFF and GRP-eGFP cells form largely non-overlapping populations, although there is some overlap between NKB/NTS and CCK/substance P populations. Protein kinase Cγ (PKCγ) is expressed by many of the NTS, and some of the NKB and CCK cells. The pie chart is based on quantitative data from the present study and from previous studies (Gutierrez-Mecinas et al., 2016, Gutierrez-Mecinas et al., 2019, Dickie et al., 2019).

We have previously estimated that the other neurochemical populations that we have identified (those that express neurokinin B, neurotensin, CCK, substance or GRP-eGFP) account for around two-thirds of the excitatory neurons in laminae I-II (Gutierrez-Mecinas et al., 2016, Gutierrez-Mecinas et al., 2019, Dickie et al., 2019). With our finding that the NPFF population accounts for ~ 6% of excitatory neurons, this brings the total that can be assigned to one of these populations to ~ 75% of these cells (Fig. 8).

### The role of NPFF

Neuropeptide FF was initially isolated from bovine brainstem and characterised by Yang et al. (1985). The gene encoding the precursor protein pro-NPFF was subsequently identified and sequenced, and shown to code for both NPFF and an extended peptide known as neuropeptide AF (Perry et al., 1997). Initial studies demonstrated that intracerebroventricular injection of NPFF suppressed morphine analgesia (Yang et al., 1985). However, Gouarderes et al. (1993) reported that intrathecal NPFF caused a prolonged increase in both tail-flick latency and paw pressure threshold in rats, corresponding to an analgesic effect. In addition, NPFF enhanced the analgesic action of intrathecal morphine. A subsequent study suggested that the anti-nociceptive action of spinal NPFF involved both μ and δ opioid receptors, since it was reduced by co-administration of specific antagonists acting at both of these classes of opioid receptors, while sub-effective doses of NPFF analogues enhanced the effect of both μ and δ opioid agonists administered intrathecally (Gouarderes et al., 1996).

Two G protein-coupled receptors for NPFF have been identified, and named NPFF-R1 and NPFF-R2 (Bonini et al., 2000, Elshourbagy et al., 2000, Hinuma et al., 2000). These both couple to G proteins of the Gi family (Ayachi and Simonin, 2014). NPFF-R2 is highly expressed in the spinal dorsal horn, as shown by both *in situ* hybridisation (Liu et al., 2001) and RT-PCR (Bonini et al., 2000, Yang and Iadarola, 2003), and the mRNA is also present in dorsal root ganglia (Bonini et al., 2000). This suggests that NPFF released from excitatory interneurons acts on NPFF-R2 expressed by both primary afferents and dorsal horn neurons (Yang et al., 2008). Primary afferents with mRNA for NPFF-R2 include those that express TRPM8 or MrgA3 (which are likely to be cold-sensitive and pruritoceptive, respectively) as well as peptidergic nociceptors (Zeisel et al., 2018). The anti-nociceptive action of NPFF is thought to involve opening of voltage-dependent potassium channels in primary afferent neurons (Mollereau et al., 2011). However, little is apparently known about the types of dorsal horn neuron that express the receptor. Binding sites for NPFF have also been identified in human spinal cord, with the highest levels in the superficial dorsal horn (Allard et al., 1994). This suggests that NPFF may also modulate nociceptive transmission in humans.

There is also evidence that expression of both NPFF and the NPFF-R2 are up-regulated in inflammatory, but not neuropathic, pain states (Kontinen et al., 1997, Vilim et al., 1999, Yang and Iadarola, 2003), and it has been suggested that this may contribute to the enhanced analgesic efficacy of morphine in inflammatory pain (Hylden et al., 1991, Yang et al., 2008).

### Function of Npff cells

Häring et al. (2018) used expression of the immediate early gene Arc (which codes for activity-regulated cytoskeleton-associated protein) to assess activation of their neuronal populations in response to noxious heat and cold stimuli. They reported that cells belonging to the Glut9 cluster (which correspond to the NPFF-expressing excitatory interneurons) could upregulate Arc following both types of stimulus, with ~ 10% of these cells showing increased Arc mRNA after noxious heat. Our findings extend these observations, by showing that many NPFF cells were pERK-positive (and therefore activated), not only following noxious heat, but also after other noxious (pinch, capsaicin) and pruritic (histamine, chloroquine) stimuli. We have previously reported that among all neurons in laminae I-II, between 20 and 37% show pERK-immunoreactivity with the different stimuli that were used in this study (Table 3) (Bell et al., 2016, Dickie et al., 2019). Comparison with the results for the NPFF cells, suggests that for most of the stimuli the NPFF cells were at least as likely to show pERK as other neurons in this region, while in the case of pinch and capsaicin they appear to be more likely to be activated. Since each experiment involved only a single type of stimulus, we cannot determine whether there was convergence of different types of nociceptive, or of nociceptive and pruritoceptive inputs onto individual cells. The proportions that we identified as being pERK-positive are therefore likely to have underestimated the fraction of cells that respond to one or more of these stimuli.

Since the NPFF cells are glutamatergic interneurons, their main action is presumably through glutamatergic synapses with other dorsal horn cells. Part of the NPFF axonal plexus lies in lamina I, where it may target ALT projection cells. In addition, the NPFF axons that enter the LSN have been shown to form contacts with spinothalamic neurons in this region in the rat (Aarnisalo and Panula, 1998), although synaptic connections were not identified in that study. However, many of the NPFF axons remain in lamina II, where dendrites of projection neurons are relatively infrequent (Todd, 2010). It is therefore likely that they engage in complex synaptic circuits that transmit nociceptive and pruritoceptive information (Peirs and Seal, 2016, Huang et al., 2018). Cells in laminae IIi-III that express PKCγ are thought to form part of a polysynaptic pathway that can convey low-threshold mechanoreceptive inputs to nociceptive projection neurons in lamina I under conditions of disinhibition, and thus contribute to tactile allodynia (Lu et al., 2013). However, much less is known about the roles of excitatory interneurons in circuits that underlie acute mechanical or thermal pain, or those that are responsible for hyperalgesia in either inflammatory or neuropathic pain states.

Interestingly, we found that the great majority of the NPFF cells were somatostatin-immunoreactive, and are therefore likely to have been affected in previous studies that manipulated the function of somatostatin-expressing dorsal horn neurons (Duan et al., 2014, Christensen et al., 2016). Since these studies implicated somatostatin cells in acute mechanical pain, as well as tactile allodynia in neuropathic and inflammatory pain states, our finding suggests that NPFF cells may contribute to these forms of pain. The somatostatin released by NPFF neurons that were activated by intradermal injection of pruritogens may be involved in itch, since somatostatin released from dorsal horn interneurons is thought to cause itch by a disinhibitory mechanism involving inhibitory interneurons that express the somatostatin 2a receptor (Huang et al., 2018). Assessing the roles of NPFF cells in spinal pain and itch mechanisms will require a method for selectively targeting them, presumably involving a genetically altered mouse line in which NPFF cells express a recombinase.

These results show that NPFF is expressed by a distinct population that accounts for around 6% of the excitatory interneurons in laminae I and II, and that these cells are frequently activated by noxious or pruritic stimuli.
